# Cure of ADPKD by Selection for Spontaneous Genetic Repair Events in *Pkd1*-Mutated iPS Cells

**DOI:** 10.1371/journal.pone.0032018

**Published:** 2012-02-09

**Authors:** Li-Tao Cheng, Shogo Nagata, Kunio Hirano, Shinpei Yamaguchi, Shigeo Horie, Justin Ainscough, Takashi Tada

**Affiliations:** 1 Stem Cell Engineering, Institute for Frontier Medical Sciences, Kyoto University, Kyoto Japan; 2 Department of Nephrology, Peking University Third Hospital, Beijing, China; 3 JST CREST, Kawaguchi, Saitama, Japan; 4 Department of Urology, Teikyo University, Tokyo, Japan; 5 Cardiovascular and Neuronal Remodelling, LIGHT, Leeds University, Leeds, United Kingdom; University of Southern California, United States of America

## Abstract

Induced pluripotent stem cells (iPSCs) generated by epigenetic reprogramming of personal somatic cells have limited therapeutic capacity for patients suffering from genetic disorders. Here we demonstrate restoration of a genomic mutation heterozygous for *Pkd1* (polycystic kidney disease 1) deletion (Pkd1(+/−) to Pkd1(+/R+)) by spontaneous mitotic recombination. Notably, recombination between homologous chromosomes occurred at a frequency of 1∼2 per 10,000 iPSCs. Southern blot hybridization and genomic PCR analyses demonstrated that the genotype of the mutation-restored iPSCs was indistinguishable from that of the wild-type cells. Importantly, the frequency of cyst generation in kidneys of adult chimeric mice containing Pkd1(+/R+) iPSCs was significantly lower than that of adult chimeric mice with parental Pkd1(+/−) iPSCs, and indistinguishable from that of wild-type mice. This repair step could be directly incorporated into iPSC development programmes prior to cell transplantation, offering an invaluable step forward for patients carrying a wide range of genetic disorders.

## Introduction

**Figure 1:**
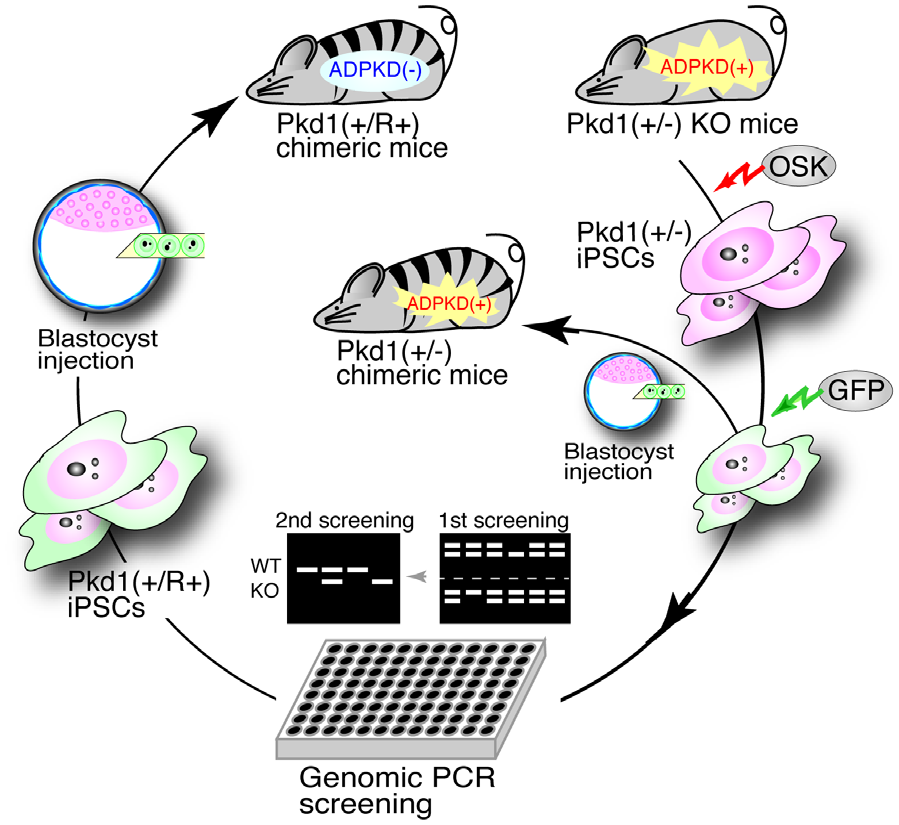
Scheme of in vitro screening and in vivo assay of mutation-restored Pkd1(+/R+) iPSCs. The knockout (KO) allele of *Pkd1* is spontaneously replaced with the wild-type allele through mitotic recombination occurring in rounds of cell division of Pkd1(+/−) iPSCs. Phenotype of ADPKD (autosomal dominant polycystic kidney disease) caused by the mutation of *Pkd1* is detected in kidneys from adult chimeric mice with Pkd1(+/−), but not Pkd1(+/R+), iPSCs.

Epigenetic reprogramming of personal somatic cells into iPSCs by forced expression of defined transcription factors confers pluripotency [1]–[3], but does not restore mutations that cause genetic disorders. For therapeutic treatment of genetic disorders, techniques using plasmid, zinc finger nucleases and helper-dependent adenoviral vector-mediated homologous recombination have been developed to induce genetic editing in disease-specific iPSCs and embryonic stem cells (ESCs) [4]–[9]. The requirement for target gene-specific vectors coupled with inherent low efficiency has restricted translation of the theoretical possibilities to practical application of these techniques in iPSCs for clinical therapy. Furthermore, Genetic modification in iPSCs gives an obstacle to safety in clinical applications. Mitotic recombination, which functions in DNA repair [10], occurs at a low frequency (<10^−6^) in somatic cells [11]. However, in experiments to drive targeted chromosome elimination from pluripotent ES-somatic hybrid cells [12], evidence suggested the frequency of genetic repair events through spontaneous mitotic recombination in pluripotent stem cells is higher than that in somatic cells [11]. Furthermore, chromosome-specific loss of heterozygosity was created in a KO allele by high-dose G418 selection to the *Neo* gene in mouse embryonic stem cells [13]. Thus, we investigated whether it was possible to identify and propagate isogenic clones of iPSCs, which retain the property of infinite cell proliferation, in which spontaneously genetic correction had occurred at disease-related mutation alleles through mitotic recombination (Figure 1).

Here, to demonstrate proof of principle for this theoretical approach, we have investigated a prevalent inherited disorder, autosomal dominant polycystic kidney disease (ADPKD). ADPKD, which is caused by genetic mutation of the *PKD1* and *PKD2* gene in 85% and 15% of cases, respectively [14], is clinically diagnosed by intrarenal cystogenesis caused by complex mechanisms. Deregulation of *PKD1* or *PKD2*-coding polycystin protein level results in initiation of cyst formation, followed by several signaling pathways that mediate cyst growth and expansion [15].

## Results and Discussion

**Figure 2:**
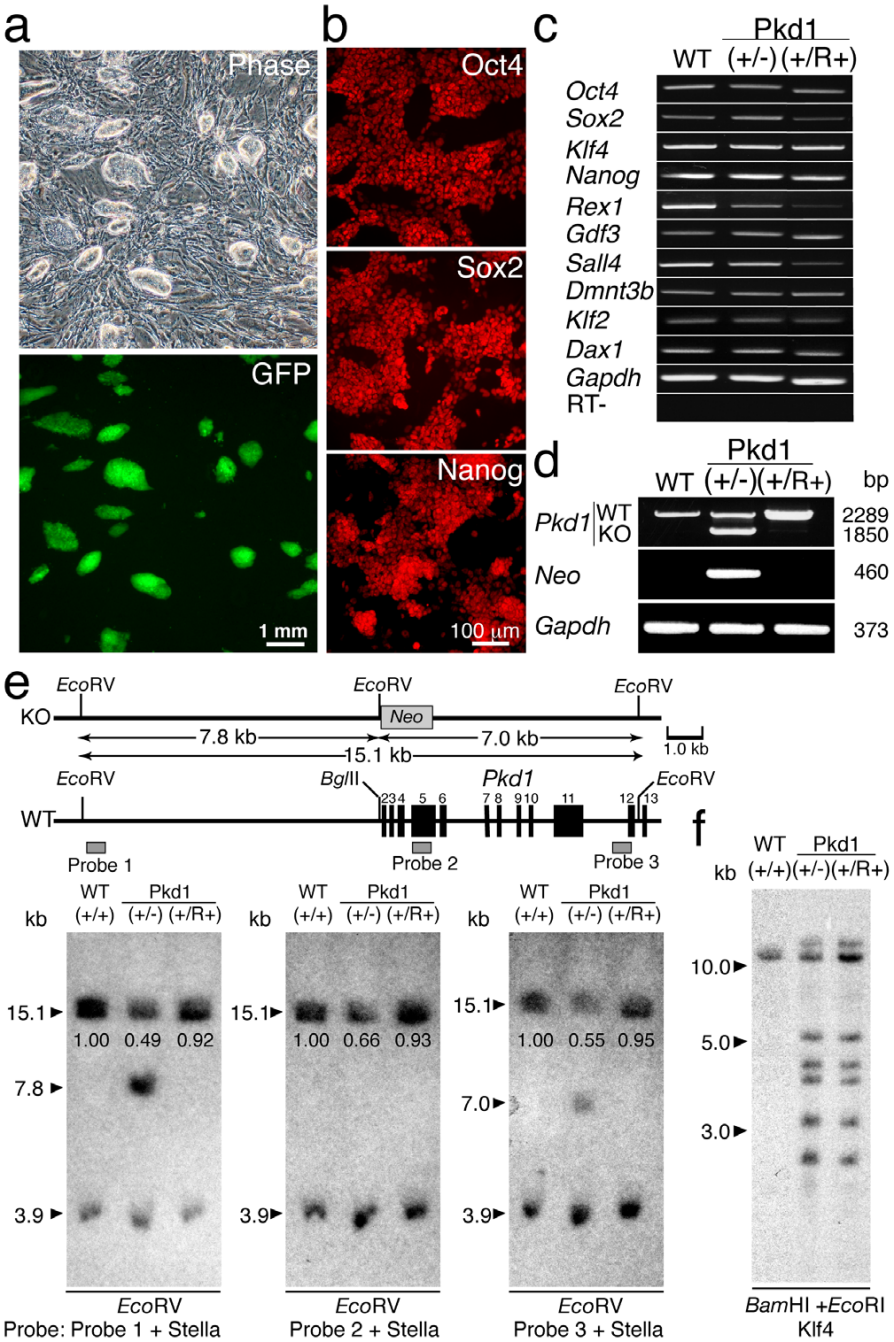
Screening of mutation-restored (Pkd1(+/R+)) iPSCs from iPSCs heterozygous for *Pkd1* knockout (KO) (Pkd1(+/−)). **a,** Normal colony morphology of Pkd1(+/−) iPSCs expressing fluorescence marker protein, GFP. **b,** Expression of pluripotent marker proteins, Oct4, Sox2, and Nanog in Pkd1(+/R+) iPSCs by immunohistochemical analyses. **c,** Transcription of pluripotent marker genes in Pkd1(+/−) and Pkd1(+/R+) iPSCs by RT-PCR analyses. **d,** Secondary screening of Pkd1(+/R+) iPSCs by genomic PCR analyses. **e,** Verification of replacement of the KO allele by the wild-type (WT) allele through spontaneous mitotic recombination in Pkd1(+/R+) iPSCs by Southern blot hybridization analyses. Relative intensity is noted under the 15.1 kb band. **f,** Determination of the origin of Pkd1(+/R+) iPSCs by Southern blot hybridization.

iPSC lines were generated from mouse embryonic fibroblasts (MEFs) derived from E12.5 *Pkd1* knockout (KO)-heterozygous embryos [16], through retroviral transduction of *Oct4*, *Sox2*, and *Klf4* (OSK) (Figure 1). The embryos were obtained by mating C57BL/6 wild-type (+/+) mice with C57BL/6 *Pkd1*(+/−) mice. Male *Pkd1*(+/−) MEFs were selected by genomic PCR analysis of the Y-chromosome-specific *Zfy* gene prior to OSK viral transfection (Figure S1). Male iPSC lines, which were re-cloned following GFP transfection, retained normal colony morphology and stable expression of GFP marker (Figure 2a). Normal karyotype, 2*n* = 40XY, was observed in 93 out of 104 (89.4%) cells examined (Figure S2). Pluripotency of Pkd1(+/−) iPSCs was validated by expression of pluripotent marker proteins, Oct4, Sox2, and Nanog by immunohistochemistry (Figure 2b), and transcription of pluripotent marker genes, *Oct4*, *Sox2*, *Klf4*, *Nanog*, *Rex1*, *Gdf3*, *Sall4*, *Dnmt3b*, *Klf2*, and *Dax1* by RT-PCR (Figure 2c). Colonies amplified from single Pkd1(+/−) iPSCs by plating sparsely into 10 cm dishes were picked, expanded and screened by genomic PCR using a *Pkd1*-specific primer set (Figure 1). Candidate clones were secondary screened by genomic PCR with an additional primer set specific to the KO allele-specific *Neo* gene (Figure 2d). In total, more than 10,000 independent colonies were screened by genomic PCR. Interestingly, two Pkd1(+/R+) clones lacking the KO allele (Figure 2d), and one Pkd1(R−/−) clone lacking the wild-type allele were detected. The frequency of spontaneous mitotic recombination-mediated mutation repair event between homologous chromosomes was estimated at 1.94×10^−4^ for Pkd1(+/R+) clone and 0.97×10^−4^ for Pkd1(R−/−) clone (Table 1). The spontaneous repair events between homologous chromosomes occurred at the frequency, which could be applicable to clinical applications.

**Table 1:** Frequency of spontaneous mitotic recombination at *Pkd1* KO allele in iPSCs.

No. of colonies			
Picked up	PCR analyzed (%)	Pkd1(+/R+) (Frequency)	Pkd1(R−/−) (Frequency)
11,232	10,322 (91.9)	2 (1.94×10^−4^)	1 (0.97×10^−4^)

To address the mechanism of allelic exchange at the *Pkd1* locus, DNA extracted from Pkd1(+/R+) iPSC lines was analyzed by Southern blot hybridization with probe 1, 2, and 3 (Figure 2e). Fragments for the KO allele, 7.8 kb with probe 1 and 7.0 kb with probe 3, were detected in Pkd1(+/−) iPSCs but not wild-type or Pkd1(+/R+) iPSCs, confirming loss of the *Neo* containing region. The intensity of the 15.1 kb band for the wild-type allele, detected by all three probes, was similar between wild-type and Pkd1(+/R+) iPSCs, and reduced by approximately 50% in Pkd1(+/−) iPSCs, when normalized against an internal loading control *Stella*, demonstrating replacement of the KO allele with the wild-type allele through mitotic recombination. To exclude the possibility of wild-type cell contamination as a source of apparent Pkd1(+/R+) iPSCs, Southern blot hybridization was performed with a probe specific to *Klf4* integrated as a multi-copy transgene in the iPSC genome but not the wild-type genome (Figure 2f). Detection of the same pattern of multiple bands in Pkd1(+/−) and Pkd1(+/R+) iPSCs demonstrates that Pkd1(+/R+) iPSCs originated from Pkd1(+/−) iPSCs.

**Figure 3:**
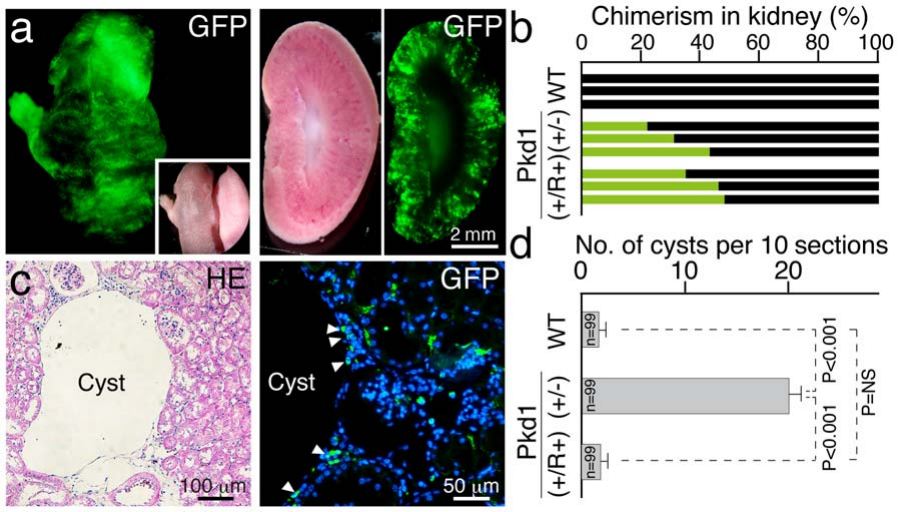
Restoration of ADPKD phenotype in adult chimeric mice with Pkd1(+/R+) iPSCs. **a,** Newborn chimeric mouse (green) generated by micro injection of Pkd1(+/−) iPSCs into host blastocyst (left panel). Contribution of Pkd1(+/R+) iPSCs into a chimeric kidney is visualized by GFP expression (right panel). **b,** Contribution of iPSCs into chimeric kidneys. Degree of iPSC contribution to each kidney collected from different chimeric mouse with Pkd1(+/−), or Pkd1(+/R+) iPSCs is indicated by a green bar. **c,** A hematoxylin-eosin (HE) section of Pkd1(+/−) chimeric kidney. A cyst was recognized as diameter greater than 200 µm (left panel). Pkd1(+/−)iPSC-derived cells (green) are located on the cyst wall (white arrow heads in right panel). Nuclei are stained as blue by DAPI. **d,** Frequency of cyst generation in kidneys from Pkd1(+/−), or Pkd1(+/R+) iPSC chimeric mice. The number of cysts was counted on HE sections of kidneys, in which degree of chimerism was estimated by GFP analysis in **b** (33 sections from each kidney). Error bars, s.e.m.

To investigate the influence of mitotic recombination on *Pkd1* expression, mRNA levels were analyzed by RT-PCR (Figure S3). Expression in Pkd1(+/−) iPSCs was approximately half that in WT or Pkd1(+/R+) iPSCs. Thus, expression of *Pkd1* was restored to the normal level in Pkd1(+/R+) iPSCs. Next, to examine physiological effects of the restoration of the KO to wild-type allele, chimeric mice were generated with Pkd1(+/−) and Pkd1(+/R+) iPSCs, both of which exhibit ubiquitous GFP expression (Figure 3a). Chimeras were analyzed at more than 6 months old following established criteria for cystogenesis as reported previously, i.e. cyst diameter >200 µm [17]. Kidneys collected in triplicate from mice generated with wild-type, Pkd1(+/−), and Pkd1(+/R+) chimeric genotypes were sacrificed for histological analyses. Extent of chimerism (22∼48%) was comparable between Pkd1(+/−) and Pkd1(+/R+) kidneys we examined (Figure 3b). Cyst formation was frequently detected in the cortical region of the Pkd1(+/−) chimeric kidneys but not the Pkd1(+/R+) and wild-type kidneys (Figure S4). GFP-positive cells comprising part of the cyst wall were observed in Pkd1(+/−) chimeric kidneys (Figure 3c), indicating that unbalanced expression level of polycystin protein in Pkd1(+/−) kidney cells might induce to initiate the cystogensis. Notably, the frequency of cyst formation was more than ten times higher in Pkd1(+/−), than Pkd1(+/R+) chimeric kidneys (Figure 3d). The number of cysts detected in Pkd1(+/R+) chimeric kidneys was comparable to that in wild-type kidneys. These data clearly demonstrate that the mutation in Pkd1(+/−) iPSCs involved in ADPKD was genetically and functionally restored in Pkd1(+/R+) iPSCs through spontaneous mitotic recombination. It is evident that selection of genetic mutation-restored iPSCs by spontaneous mitotic recombination is a realistic way to isolate isogenic and functionally restored iPSCs safely for using clinical applications.

Our results establish a paradigm for enrichment of iPSC clones, by large-scale screening, in which restoration of genetic disorder-inducing mutations has occurred spontaneously through mitotic recombination. The frequency of spontaneous mitotic recombination may not be estimated properly in pluripotent stem cells and embryonic cells in vivo and in vitro, since the event occurring in a tiny number of cells was detected using a G418-resistant selection with the *Neo* gene in many cases. In fact, mouse ESCs homozygous for the *Neo*-tagged chromosomes were selected and expanded from ESCs heterozygous for *Neo*-tagged chromosome by the high dose treatment of G418 at the frequency of approximately 1×10^−6^ [12]–[13]. The generation frequency of G418-resistant cells may be underestimated to that of homologous mitotic recombination, since some pluripotent stem cells homozygous for *Neo*, expressing insufficient amount of gene product, could stop growing or be killed. FACS analysis of mouse ESCs heterozygous for the *GFP* reporter gene without drug selection detected GFP-negative cells at the frequency of about 1×10^−3^ (data not shown), which were generated by homologous mitotic recombination or deletion of the *GFP* gene. The circumstance evidence supports that mitotic homologous recombination at an allele occurs at the frequency of about 1×10^−4^∼10^−5^, similar to our findings using iPSCs. If so, the genetic information may diverge between somatic cells thorough cell divisions rather than we predicted.

An automatic genome-sorting system could make screening of mitotic recombination-mediated genetic correction feasible. Importantly, this approach dispenses with requirement for gene therapy. As with mutation-dominant diseases such as ADPKD and LEOPARD syndrome [18], the approach is similarly applicable to genetic mutations in recessive diseases [19]. Once generated, the disease-repaired iPSCs may be useful for regenerative medicine as well as other applications. Thus, the mitotic recombination-mediated genetic correction approach will open a new path to clinical application for human iPSCs that is relevant to patient groups, for which the iPSC technological revolution was believed to hold little relevance in the clinical setting.

## Materials and Methods

### Ethics statement

Experiments with mice were performed according to the institutional guideline of Kyoto University, Japan. Our animal experiments (W-3-6) are reviewed and permitted by the animal research committee of Kyoto University, JAPAN.

### Cell culture

Pkd1(+/−) iPSC lines were established from male MEFs of E12.5 *Pkd1* knockout mouse embryos [16] by retroviral transduction of mouse OSK [20]. iPSC lines were maintained in mES medium (DMEM/F12 supplemented with 15% FBS, L-glutamine, penicillin-streptomycin, sodium bicarbonate, sodium pyruvate, 2-mercaptoethanol and 1000 U/ml of LIF (Chemicon, Temecula, CA, USA)). For isolating colonies originated from single cells, about 600 cells were plated into each 10 cm culture dish. The *EGFP* gene was introduced into Pkd1(+/−) iPSCs by lentiviral transduction.

### Chromosome

Cells were treated with 0.075 M KCl for 8 min at room temperature and then fixed in 3∶1 methanol∶acetic acid. Slides of chromosomes prepared by an air-drying method were stained with Hoechst 33258 (10 ng/ml) for G-banding analysis.

### Genomic and RT-PCR

PCR primers used in this study are listed in Table S1. For Genomic PCR, products were amplified with GoTaq (Promega) by 35 cycles of reactions. For RT-PCR, total RNA of cultured cells was extracted with TRIzol reagent (Invitrogen, USA). cDNA was synthesized from 500 ng of total RNA with Superscript III (Invitrogen, USA) using random hexamers following manufacturers instructions. Band intensity was measured using Image J (NIH).

### Southern blot hybridization

Genomic DNAs was digested with restriction enzyme(s), separated by electrophoresis on a 1.0% agarose gel, and transferred on a Hybond N^+^ filter by alkali blotting. The membrane was hybridized with probes labeled with ^32^P using the Megaprime DNA labeling system (Amersham) overnight at 42°C following pre-hybridization treatment. The membrane was washed twice in 2×SSC/0.1% SDS at 65°C for 30 minutes and twice in 0.1×SSC/0.1% SDS at 65°C for 15 minutes. Band intensity was measured using Image J.

### Chimera

For producing chimeric embryos, Pkd1(+/−) and Pkd1(+/R+) iPSCs ubiquitously expressing *EGFP*, were microinjected into C57BL/6J×BDF1 blastocysts. 6–18 months old chimeric mice were sacrificed for histological analyses.

### Histology

For immunocytochemistry, cultured cells were fixed with 4% PFA (paraformaldehyde)/PBS (phosphate-buffered saline) for 10 minutes at room temperature, washed with PBST (0.1% Triton X-100 in PBS), then pre-treated with blocking solution (3% BSA and 2% skimmed milk (DIFCO, USA) in PBST) at 4°C overnight. The cells were then stained with fluorescence-conjugated secondary antibodies (1∶500, Invitrogen), following immuno-reaction with primary antibodies; anti-OCT4 (1∶50, Santa Cruz Biotechnology, USA), anti-SOX2 (1∶500, Abcam, Cambridge, UK), and anti-NANOG (1∶200, ReproCELL, Japan). The cells were counterstained with DAPI (4,6-diamidino-2-phenylindole) and mounted with SlowFade light antifade kit (Invitrogen). Kidneys from 6–18 month old mice were fixed with 4% PFA/PBS for 4–6 hours, and embedded in paraffin. Sections at 5 µm in thickness were stained with haematoxylin and eosin.

## Supporting Information

Figure S1 **Genotyping of mouse embryonic fibroblasts (MEFs) from E12.5 embryos generated by mating of wild-type (WT) and Pkd1(+/−) mice.** *Zfy* is PCR product specific to male. M; male, F; female. Male MEFs (no. 2) heterozygous for *Pkd1* knockout (KO) was used for iPSC generation.
(TIF)

Figure S2 **Karyotype of Pkd1(+/R+) iPSC.** Normal karyotype, 2*n* = 40,XY is shown chromosomally.
(TIF)

Figure S3 **Transcription of Pkd1 mRNA in Pkd1(+/−) and Pkd1(+/R+) iPSCs.** Transcription level of mRNA compared by band intensity is comparable between wild-type (WT) and mutation restored Pkd1(+/R+) iPSCs, while is an approximately half of WT in Pkd1(+/−) iPSCs.
(TIF)

Figure S4 **Hematoxylin-eosin sections of kidneys at low magnification.** Cysts are frequently found in Pkd1(+/−) chimeric kidneys, but not wild-type (WT) and mutation-restored Pkd1(+/R+) chimeric kidneys.
(TIF)
