# Supplementary figures and images for: Cure of ADPKD by Selection for Spontaneous Genetic Repair Events in Pkd1-Mutated iPS Cells
Source: PLoS One. 2012 Feb 9;7(2):e32018. doi: 10.1371/journal.pone.0032018 (PMC3276537; doi:10.1371/journal.pone.0032018)

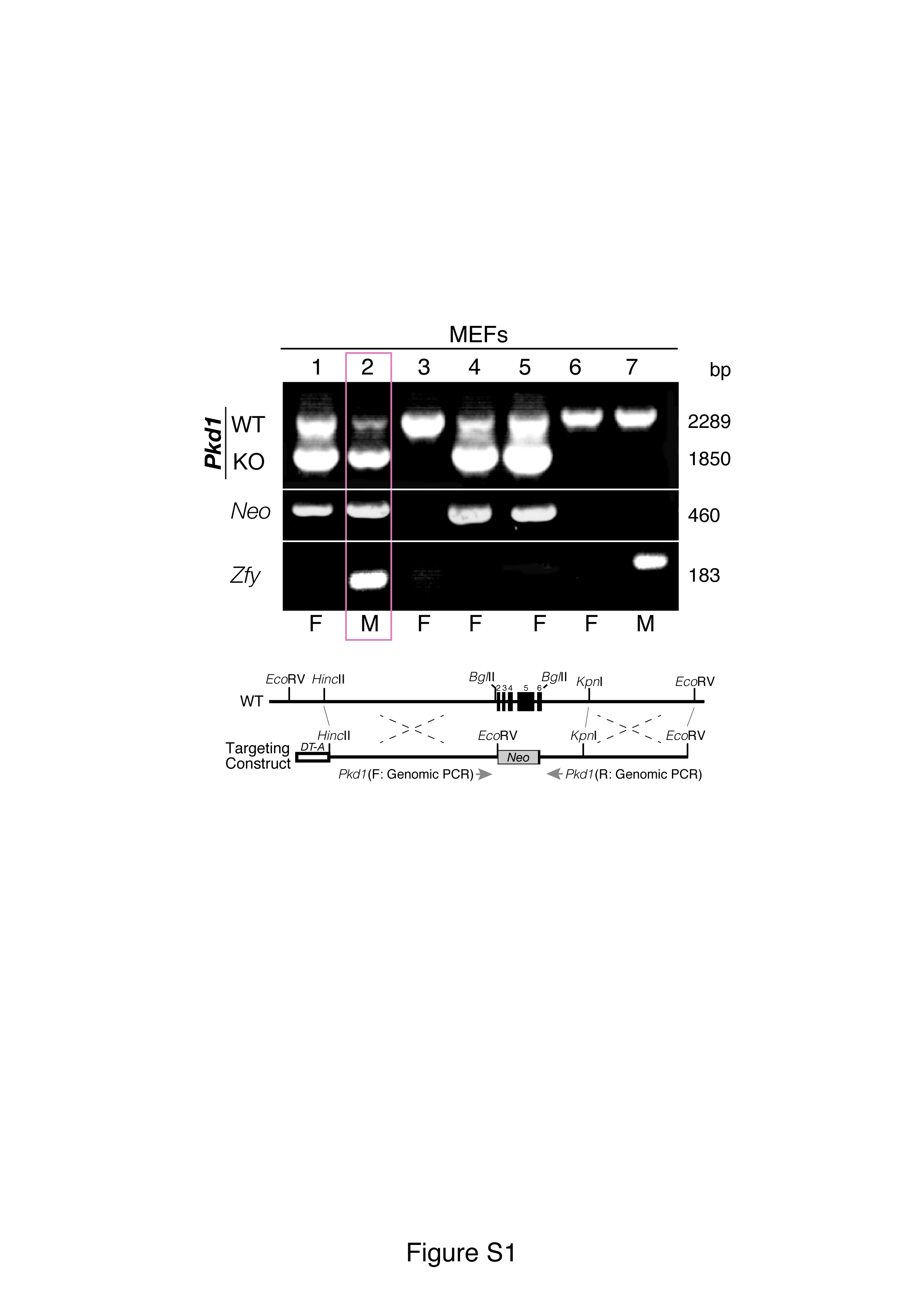

Supplement: Figure S1 — Genotyping of mouse embryonic fibroblasts (MEFs) from E12.5 embryos generated by mating of wild-type (WT) and Pkd1(+/−) mice. Zfy is PCR product specific to male. M; male, F; female. Male MEFs (no. 2) heterozygous for Pkd1 knockout (KO) was used for iPSC generation. (TIF) [file pone.0032018.s001.tif]

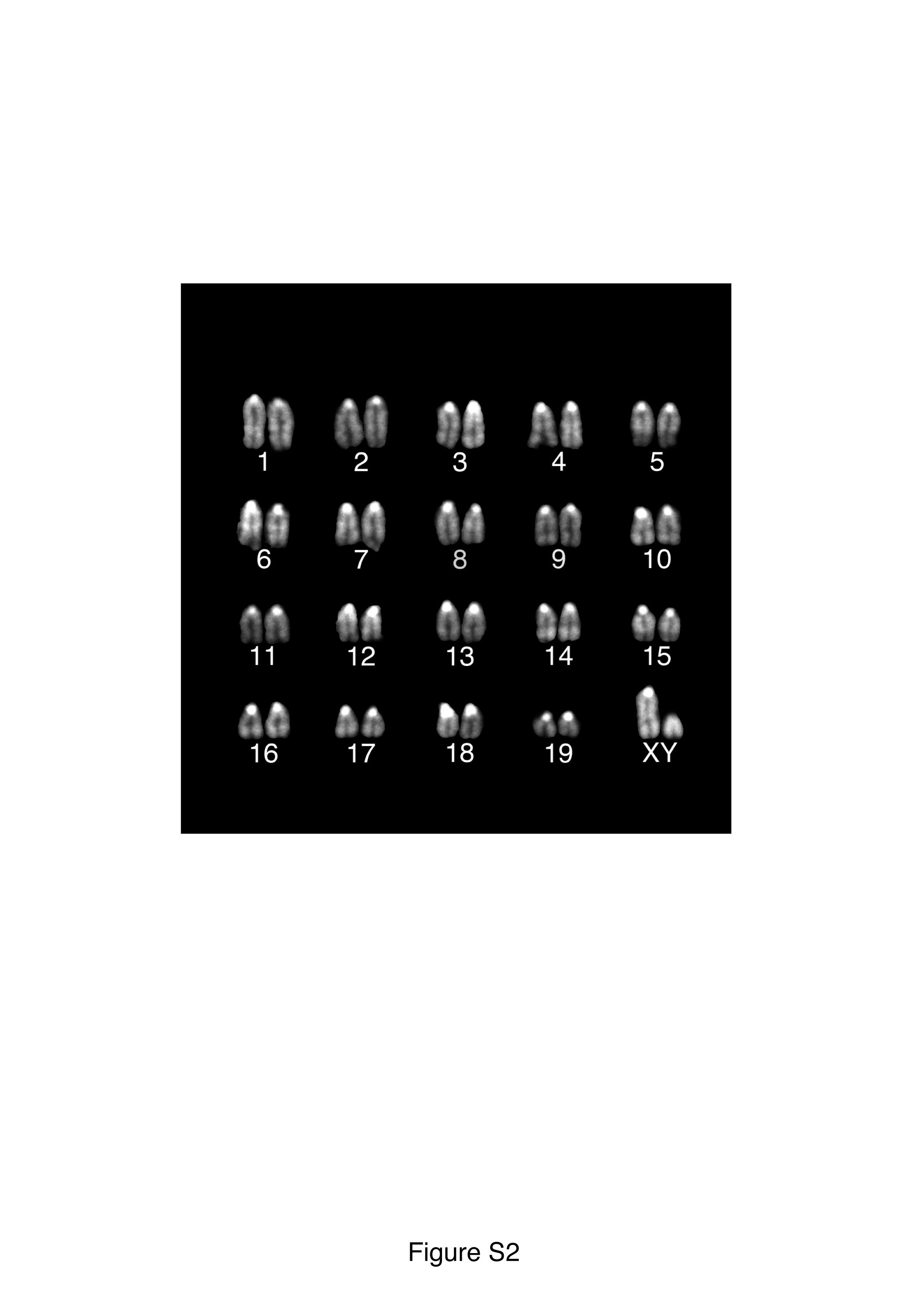

Supplement: Figure S2 — Karyotype of Pkd1(+/R+) iPSC. Normal karyotype, 2n = 40,XY is shown chromosomally. (TIF) [file pone.0032018.s002.tif]

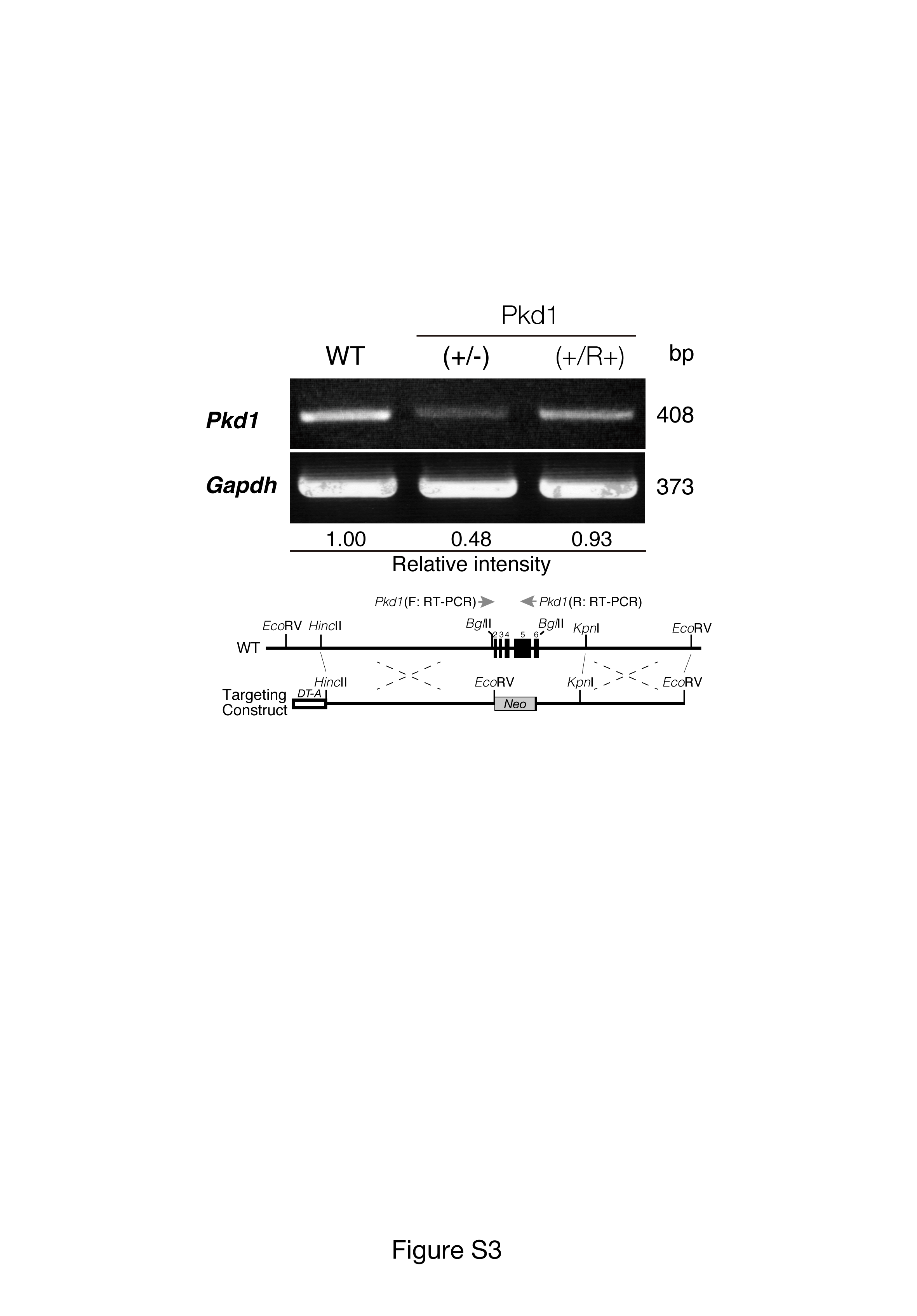

Supplement: Figure S3 — Transcription of Pkd1 mRNA in Pkd1(+/−) and Pkd1(+/R+) iPSCs. Transcription level of mRNA compared by band intensity is comparable between wild-type (WT) and mutation restored Pkd1(+/R+) iPSCs, while is an approximately half of WT in Pkd1(+/−) iPSCs. (TIF) [file pone.0032018.s003.tif]

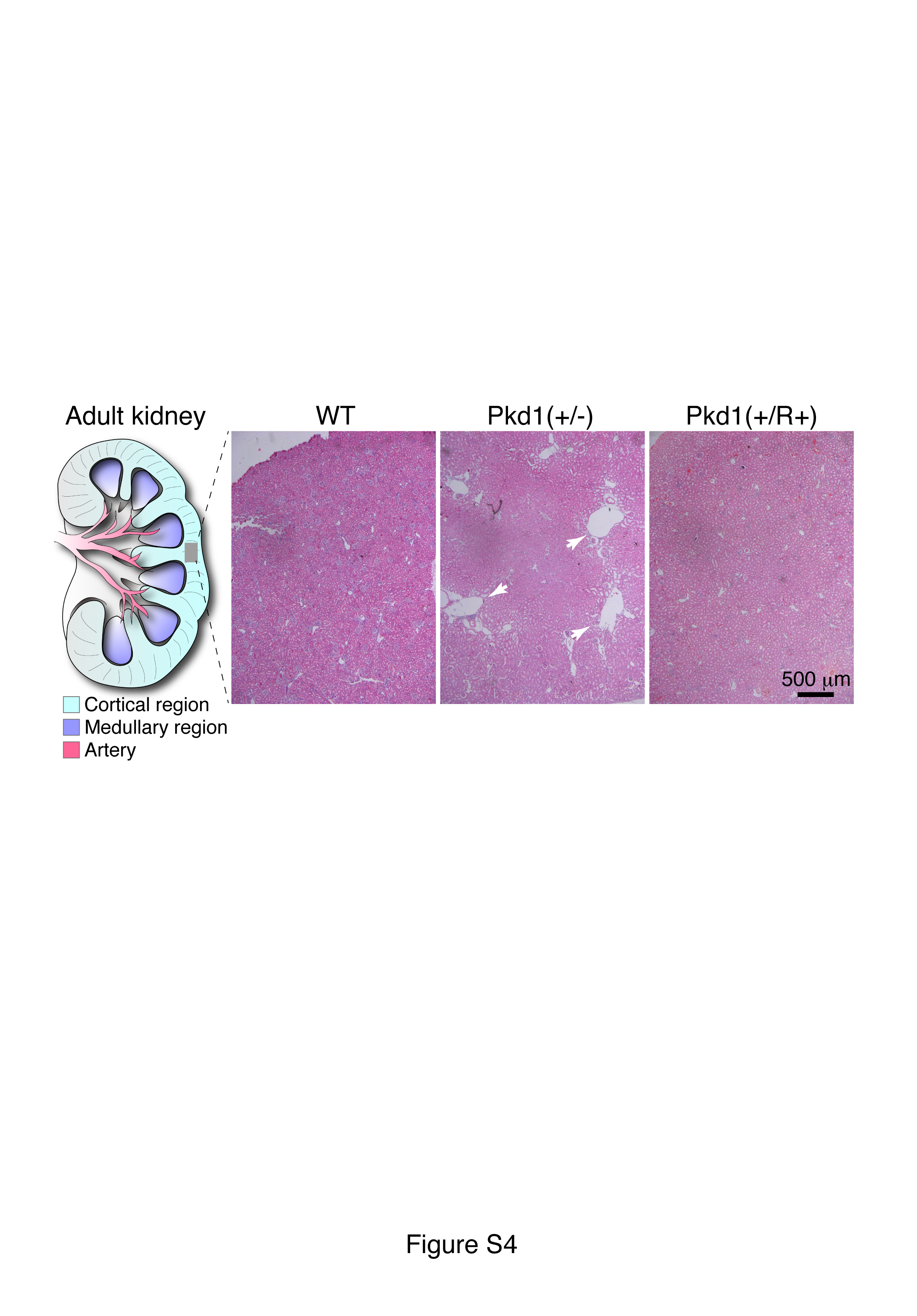

Supplement: Figure S4 — Hematoxylin-eosin sections of kidneys at low magnification. Cysts are frequently found in Pkd1(+/−) chimeric kidneys, but not wild-type (WT) and mutation-restored Pkd1(+/R+) chimeric kidneys. (TIF) [file pone.0032018.s004.tif]
